# *P. aeruginosa* Lipopolysaccharide-Induced MUC5AC and CLCA3 Expression Is Partly through Duox1 *In Vitro* and *In Vivo*

**DOI:** 10.1371/journal.pone.0063945

**Published:** 2013-05-14

**Authors:** Wen Li, Fugui Yan, Hongbin Zhou, Xiaoping Lin, Yinfang Wu, Ce Chen, Niya Zhou, Zhihua Chen, Jian-dong Li, Huahao Shen

**Affiliations:** 1 Department of Respiratory and Critical Care Medicine, Second Hospital of Zhejiang University School of Medicine, Hangzhou, Zhejiang, China; 2 Department of Respiratory and Critical Care Medicine, Second Hospital of Fujian Medical University, Quanzhou, Fujian, China; 3 Center for Inflammation, Immunity and Infection, Department of Biology, Georgia State University, Atlanta, Georgia, United States of America; 4 State Key Laboratory of Respiratory Disease, Guangzhou, China; University of Houston, United States of America

## Abstract

**Background:**

We have previously found that reactive oxygen species (ROS) are involved in *Pseudomonas aeruginosa* lipopolysaccharide (*PA-LPS*) induced MUC5AC in airway epithelial cells. Dual oxidase1 (Duox1), a member of NADPH oxidase(Nox), is known to be responsible for ROS production in respiratory tract epithelial cells. Our aim was to clarify whether Duox1 was also involved in the *PA-LPS*-induced MUC5AC and calcium dependent chloride channel 3(Clca3), another recognized marker of goblet cell hyperplasia and mucus hyper-production.

**Methods:**

*PA-LPS*-induced Duox1 mRNA levels were examined in A549 cells, primary mouse tracheal epithelial cells (mTECS) and lung tissues of mice. Nox inhibitors diphenyleneiodonium chloride (DPI) and Duox1 siRNA were used to investigate whether Duox1 is involved in *PA-LPS*-induced MUC5AC and Clca3 expression both in vitro and in vivo.

**Results:**

Duox1 is induced by *PA-LPS* in A549 cells, primary mTECs and lung tissues of mice. DPI significantly inhibited *PA-LPS*-induced up-regulation of Duox1, Muc5ac and Clca3 in primary mouse trachea epithelial cells and lung tissues of mice. Knockdown of Duox1 markedly inhibited *PA-LPS*-induced MUC5AC expression via a ROS-TGF-α cascade in A549 cells. Furthermore, DPI significantly inhibited *PA-LPS*-induced increases in inflammatory cells accumulated in mouse lungs.

**Conclusions:**

We demonstrate for the first time that *PA-LPS*-induced MUC5AC and Clca3 expression is partly through Duox1, and provide supportive evidence for Duox1 as a potential target in treatments of mucin over-production diseases.

## Introduction

Mucus hypersecretion is commonly observed in chronic inflammatory airway diseases such as asthma, chronic obstructive pulmonary disease, and cystic fibrosis. Excessive production of mucus contributes to morbidity and mortality in these diseases by plugging the airways and causing recurrent infections [1], [2]. MUC5AC mucin is the major component of airway mucus [3], [4]. A number of in vitro and in vivo studies have been carried out to explore the signaling mechanisms underlying the regulation of MUC5AC expression induced by many different stimuli [5], [6], [7], [8], [9], [10]. *Pseudomonas aeruginosa* (*PA*) infection is common in chronic inflammatory airway diseases [11], especially in cystic fibrosis. It has been previously shown that *Pseudomonas aeruginosa* lipopolysaccharide (*PA-LPS*) significantly up-regulates MUC5AC mucin expression in airway epithelial cells [12], [13], [14]. However, the underlying molecular mechanisms remain largely unknown.

Reactive oxygen species (ROS) have been found to play important roles in cigarette smoke, neutrophil elastase and phorbol 12-myristate 13-acetate (PMA) induced MUC5AC mucin expression [15], [16], [17]. In addition, we have previously shown that ROS are involved in *PA-LPS* induced MUC5AC production [18]. Dual oxidases (Duox), members of the NADPH oxidase (Nox) family, originally identified and cloned from the epithelium of the thyroid gland [19], [20], [21], were initially found to be responsible for ROS production, which is involved in the anti-microbial activity of lactoperoxidase in respiratory tract epithelial (TBE) cells [22]. The two Duox isoforms Duox1 and Duox2 have high structural similarity. While they were differentially regulated by a variety of proinflammatory factors, Duox1 is induced by Th2 cytokines IL-4 and IL-13, whereas Duox2 is induced by Th1 cytokines IFN-γ and poly(I:C) [23]. In addition, Duox1 and Duox2 have distinct functions in airway epithelium. Duox2 is mainly involved in inflammation, whereas Duox1 is mainly responsible for mucus production [17]. Nadel et al [17] have recently reported that Duox1 mediated neutrophil elastase- and PMA-induced MUC5AC mucin expression in airway epithelial cells. However, it is still unclear if Duox1 could also be involved in LPS-induced MUC5AC mucin expression. We hypothesized that Duox1 may also mediate *PA-LPS*-induced MUC5AC expression via controlling ROS production in airway. Based on our previous finding that the ROS-TGF-α signaling pathways mediate *PA-LPS*-induced MUC5AC expression in NCI-H292 cells [18], we explored whether Duox1 controlls the production of ROS and TGF-α in airway epithelial cells. To further confirm the specific role of Duox1 in mucins regulation, we also investigated whether knockdown of Duox2 can influence MUC5AC expression in A549 cells. Furthermore, because we previously showed that ROS scavenger dimethylthiourea (DMTU) inhibited *PA-LPS*-induced MUC5AC expression in vitro, we sought to further investigate whether DMTU also plays a similar role in vivo.

Calcium dependent chloride channels (CLCA) of airway epithelial cells play an important role in the regulation of mucus production [24], [25]. The expression of human CLCA1 (hCLCA1) is increased in patients with asthma [26] and COPD [27] in which mucus was excessively produced in the airway. Similarly, the expression of calcium dependent chloride channel 3(Clca3), the mouse homolog of hCLCA1, markedly increased and was closely correlated with up-regulation of MUC5AC during mucus overproduction in the airway of mice [28], [29], and we thus investigated whether Clca3 can be also induced by *PA-LPS*, and regulated by duox1 during *PA-LPS* induced MUC5AC expression. Furthermore, because *PA-LPS*-induced MUC5AC production is often accompanied by the increased airway inflammation, we investigated whether Duox1 may also regulate the number of total inflammatory cells and neutrophils in BALF of mice treated with *PA-LPS*.

To test our hypothesis, we investigated whether Duox1 is induced by *PA-LPS* in A549 cells, primary mouse tracheal epithelial cells (mTECS) and lung tissues of mice respectively, and Nox inhibitors diphenyleneiodonium chloride (DPI) inhibits *PA-LPS*-induced Duox1, MUC5AC and Clca3 expression in vitro and in vivo. The roles of Duox1 and Duox2 in *PA-LPS*-induced MUC5AC expression were also further verified by knockdown of Duox1 and Duox2 in A549 cells using small interfering RNA (siRNA). In the present study, we demonstrated for the first time that Duox1 is partly involved in *PA-LPS*-induced MUC5AC and Clca3 expression.

## Materials and Methods

### Materials

A549 cells were purchased from the American Type Culture Collection (ATCC, Manassas, VA). Diphenyleneiodonium chloride (DPI) and dimethylthiourea (DMTU) were from Calbiochem. *PA-LPS* from serotype 10 was from Sigma.

### Animals

8–10 week-old C57BL/6 mice were purchased from the Experimental Animal Center of Zhejiang University, animal protocols and procedures were approved by the Ethical Committee for Animal Studies at Zhejiang University, China. Mice were once administered *PA-LPS* (100 µg/50 µL) by tracheal cannula. After 30 minutes of *PA-LPS* administration, mice were injected (intraperitoneal injection, i.p) with DPI (1 mg/kg) or DMTU (1 mg per mice) once a day, and sacrificed after 6 days. The BALF of mice was collected to count numbers of inflammatory cells. The lungs were fixed in 10% buffered formalin and stained with Alcian blue/periodic acid-Sehiff (AB/PAS) and Hematoxylin & Eosin (H&E) staining.

### Primay Mouse Tracheal Epithelial Cells Culture

Mouse trachea was isolated from C57BL/6 mice under sterile conditions, and digested with 10 mL 0.15% Pronase solution overnight at 4°C. Then tracheal epithelial cells were harvested and submerged-cultured with mTECS proliferation medium (DMEM/F12 basic media add HEPES, glutamine solution, NaHCO3, heat-inactivated FBS, Retinoic acid, Insulin, Epidermal growth factor solution, bovine pituitary extract, Transferrin) in transwell plates(Corning, NY) for 10–14 days. When cells were confluent, the medium in apical side was removed and air-liquid interface (ALI) culture began. After 1-week of ALI culture, cells were stimulated by adding *PA-LPS* (2.5 µg/mL) and DPI (5 µM) in the basal wells for 24 hours.

### A549 Cells Culture

A549 cells were cultured in RPMI-1640 medium supplemented with 10% (vol/vol) fetal bovine serum. Before experiments, confluent A549 cells were serum-starved for 24 h to maintain low basal levels of MUC5AC expression.

### Real-time PCR

Total RNA was isolated from cells and mice lung tissues using TRIzol Reagent (Invitrogen) according to the manufacturer’s instruction. For RT-PCR, cDNA was generated by reverse transcription using 2 µg total RNA. The expression levels of mClca3 and MUC5AC mRNA were determined by quantitative real-time PCR using the SYBR Green system (Takara) on a spectrofluorometric thermal cycler (iCycler; Bio-Rad). The PCR primers are as follows: human MUC5AC: forward: GGACTTCAATATCCAGCTACGC, reverse: CAGCTCAACAACTAGGCCATC; mouse Muc5ac: forward: GGACTTCAATATCCAGCTACGC, reverse: GGACTT CAATATCCAGCTACGC; human Duox1: forward: CCTGGCTCTAGCATG GACAC, reverse: CTGCACCTCCCACGAAATG; mouse Duox1: forward: GCGATTTGATGGATGGTAT, reverse: TAGGCAGGTAGGGTTCTTT; human Duox2: forward: AAGTTCAAGCAGTACAAGCGAT, reverse: TAGGCACGGTC TGCAAACAG; mouse Clca3: forward: ACTAAGGTGGCCTACCTCCAA, forward: GGAGGTGACAGTCAAGGTGAGA.

### RNAi

Human Duox1 and Duox2 siRNAs were purchased from Ambion (Austin, TX). SiRNA was transfected into A549 cells by using Lipofectamine 2000 (Invitrogen, # 11668019).

### Immunohistochemistry

Cells were incubated with mouse monoclonal antibody to MUC5AC (clone 45 M1, 1∶100) for 1 h followed by 10 min in biotinylated rabbit anti-mouse antibody (1∶300) and another 10 min in streptavidin–biotin horseradish peroxidase (1∶50). Cells were developed for 2 min with diaminobenzidine as chromogen substrate (DAKO Ltd.), counterstained with hematoxylin, and mounted in a xylene-based mountant(BDH-Merck, UK).

### H_2_O_2_ Measurements

Cells were treated with *PA-LPS* (10 µg/mL) for 2 h, H_2_O_2_ production in the cell supernatants was measured by using the Amplex Red Hydrogen Peroxide/Peroxidase Assay kit (Invitrogen) according to the manufacturer’s instructions.

### ELISA

After reaching confluence and being serum starved for 24 h, cells were stimulated with *PA-LPS* (10 µg/mL) for 4 h. Cell supernatants were collected and concentrated 10-fold using an Ultracel YM-3 Centrifugal Filter Device (Millipore), and the levels of TGF-α in concentrated cell supernatants were quantified using ELISA kits (R&D Systems Inc, Minneapolis, MN).

### Statistical Analysis

Data are presented as mean±SD (n = 3). ANOVA was used to determine statistically significant differences (*P*<0.05).

## Results

### *PA-LPS* Induced Duox1 Expression in Primary Mouse Trachea Epithelial Cells and in Mouse Lungs

**Figure 1:**
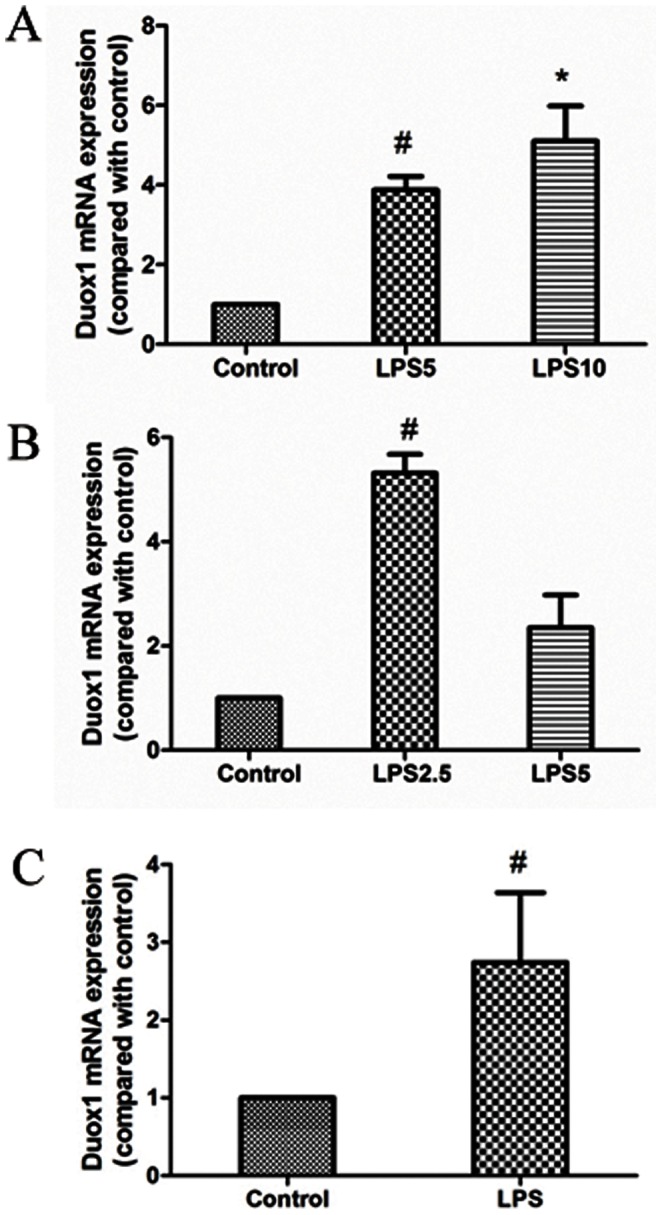
*PA-LPS* induced Duox1 expression in A549 cells, primary mouse trachea epithelial cells and in mouse lungs. A: *PA-LPS*(5 µg/mL and 10 µg/mL) significantly up-regulated Duox1 mRNA expression in A549 cells; B:*PA-LPS*(2.5 µg/mL) significantly up-regulated Duox1 mRNA expression in primary mTECs; C:*PA-LPS* (100 µg) significantly up-regulated Duox1 mRNA expression in lung tissues of mice; ^#^ *P*<0.05 compared with control,*P<0.05 compared with control.

As an initial approach to explore the possible role of Duox1 in mediating *PA-LPS* induced mucus production, the induction of Duox1 by *PA-LPS* in A549 cells, mTECs and mouse lung tissues was analyzed. As expected, *Duox1* mRNA was markedly increased in A549 cells, mTECs and mouse lungs treated with *PA-LPS* (Figures 1A–1C), though there was no dose-dependent effect for such an induction. These data suggested that Duox1 might positively regulate the mucus production in context of *PA-LPS* treatment.

### Suppression of *PA-LPS*-induced Muc5ac and mClca3 Expression by DPI through Decreasing the Expression of Duox1 in Primary Mouse Trachea Epithelial Cells

**Figure 2:**
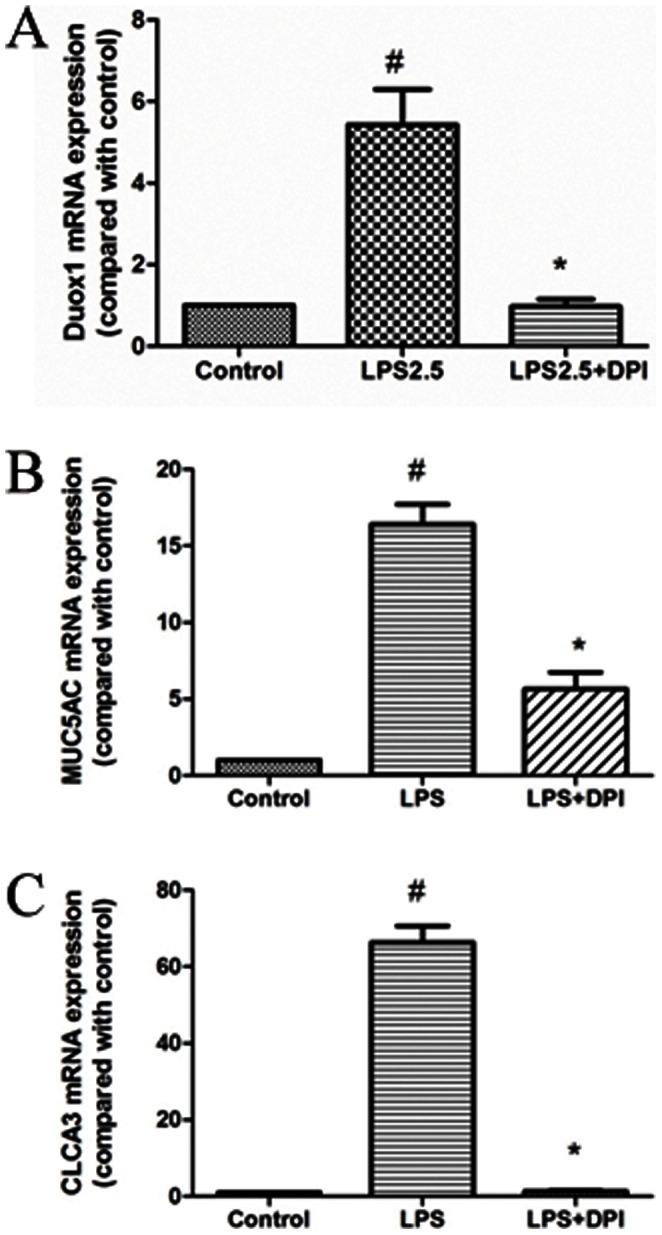
Suppression of *PA-LPS*-induced Duox1, *Muc5ac* and *Clca3* expression by DPI in primary mouse trachea epithelial cells. A: Diphenylene iodonium(2.5 µM) significantly inhibited *PA-LPS*-induced Duox1 mRNA expression in primary mTECs. B: Diphenylene iodonium(2.5 µM) significantly inhibited *PA-LPS*-incuced Muc5ac mRNA expression in primary mTECs. C: Diphenylene iodonium(2.5 µM) significantly inhibited *PA-LPS*-induced Clca3 mRNA expression in primary mTECs.^ #^ *P*<0.05 compared with control, *P<0.05 compared with *PA-LPS*.

To clarify the role of Duox1 in *PA-LPS*-induced mucus production, we utilized an NADPH oxidase inhibitor DPI which is known to inhibit the function of Duox1 [17], as genetic approaches are difficult to be used in primary epithelial cultures. As shown in Figures 2A–2C, DPI (2.5 µM) significantly inhibited the *PA-LPS* (2.5 µg/mL) induced mRNA transcripts of Duox1, and also reduced the expression of Muc5ac and Clca3 mRNA, both of which are well recognized markers of goblet cell hyperplasia and mucus hyper-production. These results showed that DPI inhibited *PA-LPS* induced-Muc5ac and Clca3 expression partly through decreasing the expression of Duox1 in primary mTECs.

### Duox1 Small Interfering RNA Inhibited *PA-LPS*-induced MUC5AC Expression in A549 Cells

**Figure 3:**
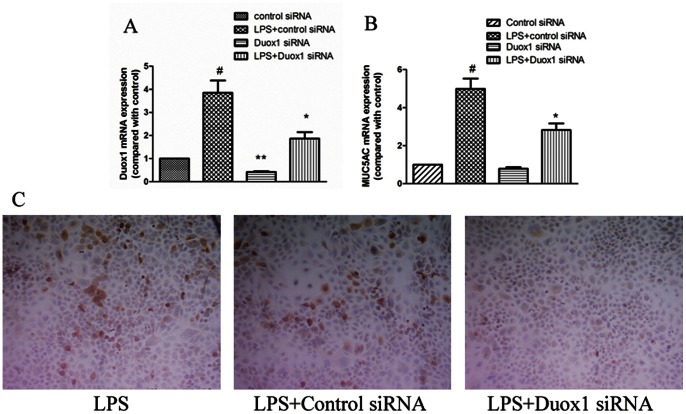
Duox1 small interfering RNA inhibited *PA-LPS*-induced *MUC5AC* expression in A549 cells. A: Duox1 siRNA(100 nM) significantly inhibited Duox1 mRNA expression in normal as well as *PA-LPS*-stimulated(10 µg/mL) A549 cells. B:Duox1 siRNA(100 nM) significantly inhibited *PA-LPS* -incuced MUC5AC mRNA expression in A549 cells; C: Duox1 siRNA(100 nM) significantly inhibited *PA-LPS*-incuced MUC5AC protein expression in A549 cells (×200). ^#^ *P*<0.05 compared with control siRNA. *P<0.05 compared with *PA-LPS*+control siRNA. **P<0.05 compared with control siRNA.

To further confirm whether Duox1 is required for *PA-LPS*-induced MUC5AC expression in airway epithelial cells, we examined the effect of Duox1 knockdown on *PA-LPS*-induced MUC5AC expression in A549 cells by real-time PCR and immunohistochemistry. As shown in Figure 3A, Duox1 siRNA(100 nM) significantly inhibited Duox1 mRNA expression in A549 cells, and knockdown of Duox1 (100 nM Duox1 siRNA) significantly reduced *PA-LPS-*induced expression of MUC5AC mRNA in A549 cells (decreased by 44% compared with control, *P*<0.05) (Figure 3B). Furthermore, using mouse monoclonal antibody to MUC5AC (clone 45 M1, 1∶100), we confirmed that 100 nM Duox1 siRNA markedly inhibited MUC5AC protein expression in A549 cells (Figure 3C). These results suggested that *PA-LPS*-induced MUC5AC expression was Duox1-dependent in airway epithelial cells.

### Duox2 Small Interfering RNA Exerted no Considerable Effects on *PA-LPS*-induced MUC5AC expression in A549 Cells

**Figure 4:**
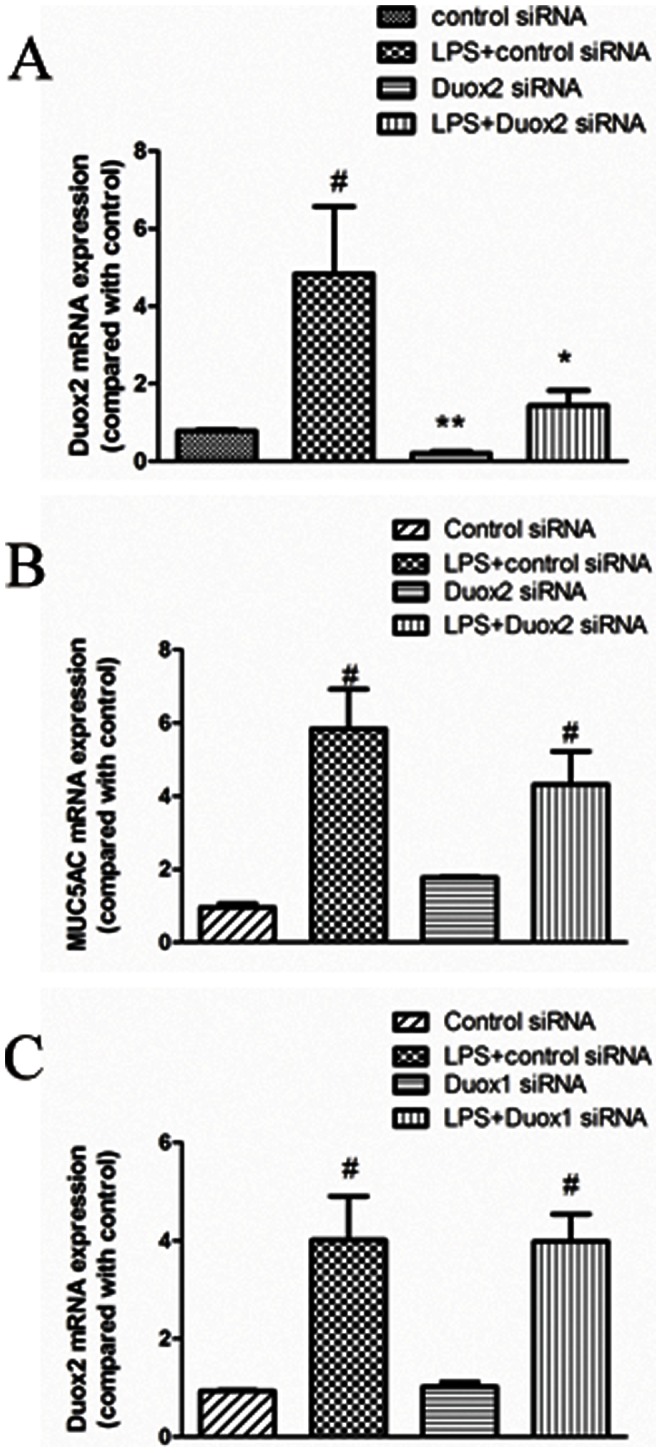
Role of Duox2 small interfering RNA in *PA-LPS*-induced MUC5AC expression in A549 cells. A: Duox2 siRNA(100 nM) significantly inhibited Duox2 mRNA expression in normal as well as *PA-LPS*-stimulated(10 µg/mL) A549 cells. B: Duox2 knockdown exerted no considerable effects on *PA-LPS* -incuced MUC5AC mRNA expression in A549 cells; C: Duox1 knockdown has no effects on Duox2 expression in normal as well as *PA-LPS*-stimulated(10 µg/mL) A549 cells. ^#^ *P*<0.05 compared with control siRNA. *P<0.05 compared with *PA-LPS*+control siRNA. **P<0.05 compared with control siRNA.

To investigate whether Duox2 is also involved *PA-LPS*-induced MUC5AC expression, we transfected Duox2 siRNA into A549 cells and showed that Duox2 siRNA could not significantly inhibit *PA-LPS*-induced MUC5AC expression (Figure 4A–4B). Furthermore, we showed that Duox1 siRNA transfection had no obvious effects on Duox2 expression (Figure 4C). These data supported that Duox2 function is different from Duox1 in airway epithelial cells, especially in term of mucin regulation.

### Duox1 is Required for *PA-LPS*-induced ROS and TGF-α Production in A549 Cells

**Figure 5:**
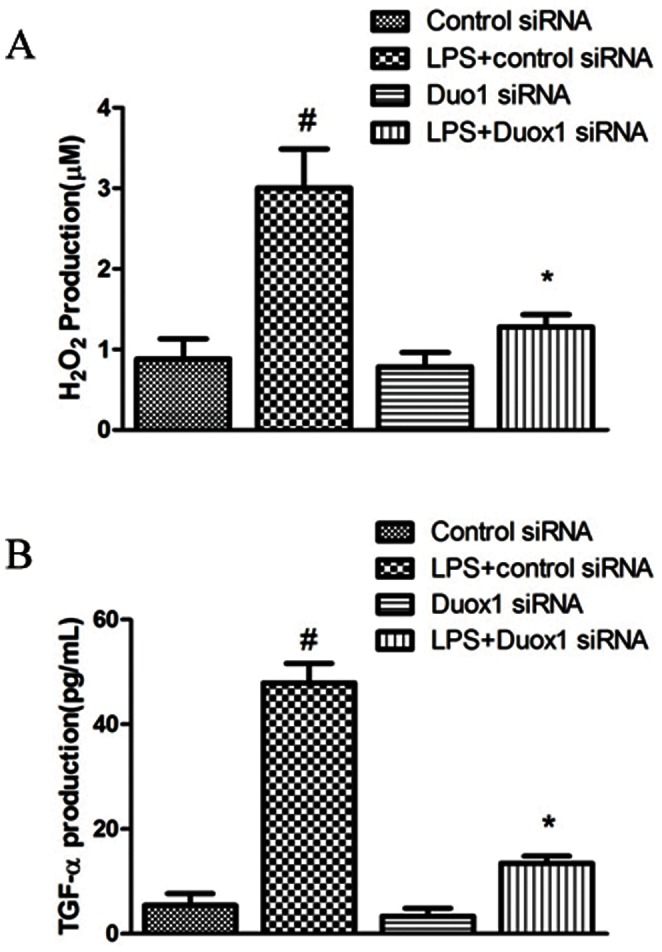
Duox1 is required for *PA-LPS*-induced ROS and TGF-α production in A549 cells. A: Duox1 siRNA(100 nM) significantly inhibited *PA-LPS*-incuced H_2_O_2_ production in A549 cells. B: Duox1 siRNA(100 nM) significantly inhibited *PA-LPS*-incuced TGF-α production in A549 cells. ^#^ *P*<0.05 compared with control siRNA. *P<0.01 compared with *PA-LPS*+control siRNA.

On the basis of our previous finding showing the involvement of the ROS-TGF-**α** cascade in *PA-LPS*-induced MUC5AC expression [18], we further investigated whether Duox1 mediates the production of ROS and TGF-**α**. As shown in Figure 5A and 5B, 100 nM Duox1 siRNA significantly reduced *PA-LPS*-induced ROS and TGF-alpha production in A549 cells. Taken together, these data demonstrated that Duox1 is involved *PA-LPS*-induced MUC5AC expression via a ROS-TGF- **α**-dependent mechanism.

### Effects of DMTU and DPI on *PA-LPS*-induced Muc5ac and mClca3 Production *in vivo*

**Figure 6:**
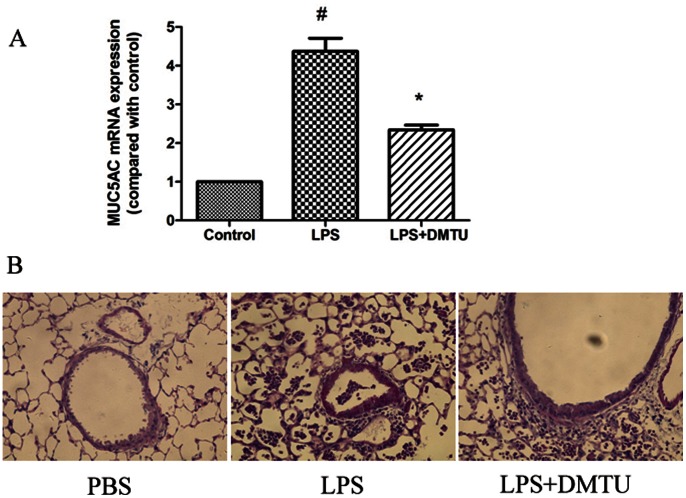
Role of ROS in *PA-LPS*-induced *Muc5ac* production in vivo. A: DMTU (1 mg per mouse) significantly blocked *PA-LPS*-induced *Muc5ac* mRNA and mucin expression in the lung of mice; B: DMTU (1 mg per mouse) inhibited *PA-LPS*-induced mucin expression in the lung of mice (PAS ×400). ^#^ *P*<0.05 compared with control, *P<0.01 compared with *PA-LPS*.

**Figure 7:**
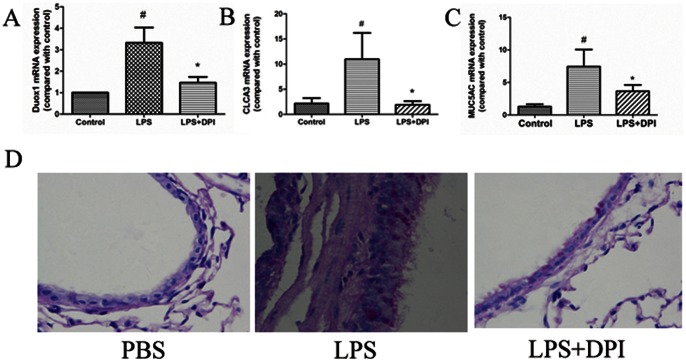
Effects of DPI on *PA-LPS*-induced Duox1, *Muc5ac* and Clca3 production in vivo. A–C: DPI (1 mg/kg) blocked *PA-LPS*-induced Duox1, Clca3 and Muc5ac mRNA expression in the lung of mice; D: DPI (1 mg/kg) inhibited *PA-LPS*-induced mucin production in the lung of mice (PAS ×400). ^#^ *P*<0.05 compared with control, *P<0.01 compared with *PA-LPS*.

We have demonstrated both ROS and Duox1 play important roles in *PA-LPS* -induced MUC5AC expression in vitro, whether same effects could exist in vivo have yet to be addressed. We previously showed that ROS scavenger DMTU significantly decreased *PA-LPS*-induced MUC5AC production in NCI-H292 cells [18]. Here we further showed that DMTU (1 mg per mouse) significantly blocked *PA-LPS*-induced Muc5ac mRNA expression in the lung tissues of mice (Figure 6A), and also inhibited mucin over-production as demonstrated by Alcian blue/periodic acid-Schiff (AB/PAS) staining (Figure 6B). As shown in figure2A–2C, DPI significantly suppressed *PA-LPS*-induced Duox1, Muc5ac and mClca3 expression in mTECs. Similarly, DPI (1 mg/kg) also inhibited *PA-LPS*-induced Duox1, Clca3 and Muc5ac mRNA, and Muc5ac mucin production in mouse lung tissues (Figure 7A–7D). These data demonstrated that Duox1 is partly involved in *PA-LPS*-induced up-regulation of Muc5ac and Clca3 in *vivo*.

### DPI Reduced *PA-LPS*-induced Inflammatory Cells in BALF and Lung Tissues of Mice

**Figure 8:**
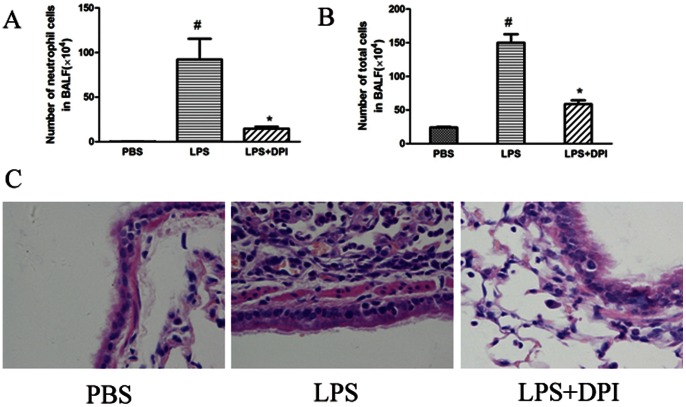
DPI reduced *PA-LPS*-induced inflammatory cells in BALF and lung tissues of mice. A–B: DPI (1 mg/kg) significantly inhibited *PA-LPS*-induced neutrophils and total cells in BALF of mice. C: DPI (1 mg/kg) significantly reduced *PA-LPS*-induced inflammatory cells in lung tissues of mice (H&E×400). ^#^ *P*<0.05 compared with PBS, *P<0.01 compared with *PA-LPS*.

*PA-LPS*-induced airway mucin over-production is usually accompanied by an increase in inflammatory cells, especially neutrophils. To determine the effects of Duox1 on *PA-LPS*-induced inflammatory cells in lung of mice, we further examined neutrophils and total inflammatory cells in BALF and lung tissues of mice treated by *PA-LPS*. As shown in figure 8A and 8B, DPI significantly reduced *PA-LPS*-induced neutrophils and total cells in BALF of mice, and also reduced inflammatory cells in lung tissues demonstrated by H&E staining (8C), thereby suggesting that Duox1 may be involved in the regulation of *PA-LPS*-induced airway inflammation in mice.

## Discussion

To the best of our knowledge, this is the first study to demonstrate that *PA-LPS*-induced up-regulation of MUC5AC and Clca3 is partly through Duox1 in vitro and in vivo. Here we showed Duox1 is induced by *PA-LPS* both in vitro and in vivo, and DPI (specific inhibitors of Nox) significantly inhibited *PA-LPS*-induced up-regulation of MUC5AC and Clca3 partly through decreasing Duox1 expression in mTECs and lung tissues of mice. In addition, we also confirmed that knockdown of Duox1 markedly inhibited *PA-LPS*-induced MUC5AC expression via a ROS-TGF-α cascade in A549 cells. Furthermore, we demonstrated that DPI significantly inhibited *PA-LPS*-induced increase in total inflammatory cells and neutrophils accumulated in BALF and lung tissues of mice. On the basis of our previous data that ROS scavengers DMTU reduced *PA-LPS*-induced MUC5AC production in NCI-H292 cells, we further confirmed that DMTU significantly inhibited *PA-LPS*-induced Muc5ac in vivo.

Initially identified and cloned from the thyroid gland, Duox are known to be expressed on the surface of ciliated airway epithelial cells [30], and Duox1 is specifically expressed in large airways [31], and its expression is 5-fold higher than that of Duox2 in normal airway epithelium [23]. Harper et al [23] found Duox1 mRNA was specially but moderately increased (by approximately four fold) by Th2 cytokines IL-4 and IL-13. Boots et al [32] showed LPS (10 µg/mL) significantly increased Duox1 mRNA expression in immortalized human bronchial epithelial (HBE1) cells. In this study we showed Duox1 mRNA was up-regulated by approximately 5-fold in vitro and 2.5-fold in vivo by *PA-LPS*. Interestingly Rada B et al [33] showed *pyocyanin* producted by *Pseudomonas aeruginosa* inhibited Duox1 activation induced by Th2 cytokines in primary normal human bronchial cells and NCI-H292 cells, suggesting the regulation of Duox1 is different in various microenvironments. Rigutto et al demonstrated activation of Duox1 is Ca^2+^ dependent [34], and whether *PA-LPS* also activates Duox1 via Ca^2+^ signaling needs to be further investigated.

Human Duox1 and Duox2 are highly similar trans-membrane proteins, while they have distinct function in airway epithelium. Duox2 is mainly involved in responses to infection and inflammation, whereas Duox1 plays an important role in defense and mucus production [17]. Nadel et al [17] confirmed PMA and neutrophil elastase-induced MUC5AC expression was Duox1-dependent in vitro. In this study we found for the first time that *PA-LPS*-induced MUC5AC production is partly through Duox1, not Duox2, thus providing supportive evidence for the role of Duox1 in mucus production in airway, and supporting that Duox2 function is different from Duox1 in airway epithelial cells, especially in term of mucins regulation. It should be noted that it is the first time to demonstrate that Duox1 was involved in mucus regulation in vivo. Kim H et al [35] demonstrated that Nox4 is involved in H_2_O_2_-induced MUC5AC over-production in normal nasal epithelial (NHNE) cells, and independent of Duox1. Thus Duox1 may be more specific for the bronchial epithelial cells in the regulation of MUC5AC expression. On the other hand, in agreement with our previous data in vitro, we showed DMTU significantly inhibit *PA-LPS*-induced MUC5AC production in vivo, further suggesting that ROS play important roles in *PA-LPS*-induced MUC5AC expression.

Clca3 is another known marker of goblet cells in mice airway, and its expression has significantly correlated with MUC5AC. Here we found Clca3 can not only be significantly induced by *PA-LPS*, but also regulated by Duox1 in *PA-LPS-*induced Muc5ac expression in mTECs and mice. In contrast to our data that Clca3 and MUC5AC could be both induce by *PA-LPS* and both regulated by Duox1, Thai P et al [36] showed that MUC5AC and Clca3 were differentially induced and regulated by IL-13 in primary tracheobronchial epithelial (TBE) cells. Thus the regulation of MUC5AC and Clca3 was distinct in response to different stimuli. In this study we confirmed the role of Duox1 in *PA-LPS*-induced MUC5AC expression in A549 cells by RNA interference, and demonstrated the function of Duox1 in mice by DPI. Its role has to be further invesigated by using Duox1-deficient mice.

Except the role of Duox1 in mucus regulation, we additionally found DPI significantly reduced neutrophils of BALF and lung tissues in mice, which suggested Duox1 may play an important role in *PA-LPS*-induced airway inflammation. Our data is in light with some previous studies. For example, Nakanaga T et al [37] showed Duox1 mediated *PA-LPS*-induced IL-8 production in airway epithelial cells, and Kong X et al [38] showed NADPH oxidase-deficiency (Gp^91phox−/−^ mice) significantly reduced LPS-induced sepsis in Nrf2^−/−^ mice. However, Zhang WJ et al [39] demonstrated genetic deficiency of NADPH oxidase does not diminish, but rather enhances LPS-induced acute inflammatory responses in mice injected ip with 50 µg LPS, and Gao XP et al [40] showed NADPH oxidase-deficiency (p^47phox−/−^ and Gp^91phox−/−^ mice) significantly increased neutrophils infiltration in lung tissues of mice injected ip by *E. coli* (2×10^8^ live *E. coli*/100 µL). One of the possible reasons for the discrepancy between these studies may be due to different animal models and protocols that were used.

### Conclusions

In summary, we firstly demonstrated that *PA-LPS*-induced MUC5AC and Clca3 expression are partly through Duox1 in vitro and in vivo, and provide supportive evidence for Duox1 as a potential target in treatments of mucin over-production diseases.
